# Developmental shift to mitochondrial respiration for energetic support of sustained transmission during maturation at the calyx of Held

**DOI:** 10.1152/jn.00333.2021

**Published:** 2021-08-25

**Authors:** Brendan J. Lujan, Mahendra Singh, Abhyudai Singh, Robert B. Renden

**Affiliations:** ^1^Department of Physiology and Cell Biology, University of Nevada, Reno School of Medicine, Reno, Nevada; ^2^Electrical & Computer Engineering, University of Delaware, Newark, Delaware

**Keywords:** bioenergetics, glycolysis, mouse, oxidative phosphorylation, synaptic vesicle cycle

## Abstract

A considerable amount of energy is expended following presynaptic activity to regenerate electrical polarization and maintain efficient release and recycling of neurotransmitter. Mitochondria are the major suppliers of neuronal energy, generating ATP via oxidative phosphorylation. However, the specific utilization of energy from cytosolic glycolysis rather than mitochondrial respiration at the presynaptic terminal during synaptic activity remains unclear and controversial. We use a synapse specialized for high-frequency transmission in mice, the calyx of Held, to test the sources of energy used to maintain energy during short activity bursts (<1 s) and sustained neurotransmission (30–150 s). We dissect the role of presynaptic glycolysis versus mitochondrial respiration by acutely and selectively blocking these ATP-generating pathways in a synaptic preparation where mitochondria and synaptic vesicles are prolific, under near-physiological conditions. Surprisingly, if either glycolysis or mitochondrial ATP production is intact, transmission during repetitive short bursts of activity is not affected. In slices from young animals before the onset of hearing, where the synapse is not yet fully specialized, both glycolytic and mitochondrial ATP production are required to support sustained, high-frequency neurotransmission. In mature synapses, sustained transmission relies exclusively on mitochondrial ATP production supported by bath lactate, but not glycolysis. At both ages, we observe that action potential propagation begins to fail before defects in synaptic vesicle recycling. Our data describe a specific metabolic profile to support high-frequency information transmission at the mature calyx of Held, shifting during postnatal synaptic maturation from glycolysis to rely on monocarboxylates as a fuel source.

**NEW & NOTEWORTHY** We dissect the role of presynaptic glycolysis versus mitochondrial respiration in supporting high-frequency neurotransmission, by acutely blocking these ATP-generating pathways at a synapse tuned for high-frequency transmission. We find that massive energy expenditure is required to generate failure when only one pathway is inhibited. Action potential propagation is lost before impaired synaptic vesicle recycling. Synaptic transmission is exclusively dependent on oxidative phosphorylation in mature synapses, indicating presynaptic glycolysis may be dispensable for ATP maintenance.

## Graphical abstract

**Figure:**
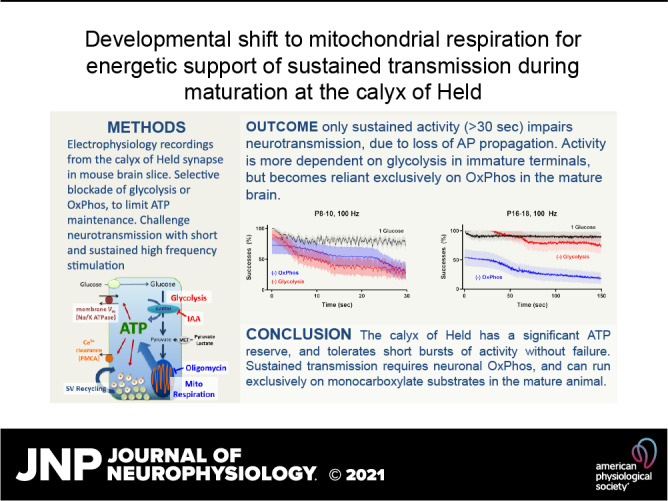

## INTRODUCTION

In neurons, glycolysis results in low-yield ATP production and provides lactate for high-yield mitochondrial ATP production via oxidative phosphorylation (OxPhos), a slower but vastly higher yield reaction requiring oxygen. Mitochondrial OxPhos is the major producer of ATP in the brain (1), and glucose feeds OxPhos to maintain presynaptic ATP levels during activity (2). However, glycolysis upregulation is uncoupled from oxygen consumption in vivo in response to abrupt increases in synaptic activity (3, 4). Highly regulated, fast glycolysis may be thus a strategy to meet immediate energy needs at synapses during the onset of activity (5). At the synapse, energy is required to fuel ion transport to maintain a hyperpolarized membrane potential at rest and to reestablish these concentration gradients after a bout of activity. Synaptic vesicle (SV) recycling is an additional major consumer of presynaptic ATP during synaptic activity at excitatory synapses. Though these reactions are conserved and well defined, conditions under which glycolysis versus mitochondrial respiration may be selectively used to support presynaptic function are still unclear.

Glycolysis and OxPhos may have functionally independent roles at the synapse. A specific role for neuronal glycolysis has been shown for several presynaptic processes: loading transmitter in vesicles (6), SV transport (7), SV localization at release sites (8), and membrane polarization (9, 10). Glycolysis can support low-frequency activity at excitatory glutamatergic synapses (11–14). During repetitive activity, disruption of mitochondrial respiration impairs SV recycling and synaptic function, but these defects are partially offset by glycolysis (15, 16). Conversely, in cases where glucose is limiting, circulating monocarboxylate substrates (lactate, pyruvate) are used to fuel OxPhos in support of transmission and neuronal survival (15, 17–19).

Less information is available regarding specific routes of energetic support for ionic pumps that facilitate transmission in neurons, but glycolysis may be selectively used. For example, Na/K ATPases are needed to maintain action potential (AP) propagation, ATP-sensitive K^+^ channel may help set membrane potential, and intracellular Ca^2+^ extrusion by plasma membrane ATPase acts to shape synaptic release probability. Glycolysis regulates many of these channels and ion transporters in other tissues (20), and glycolytic machinery is localized to presynaptic terminal membranes (21). Our previous publication showed that glycolysis shapes the presynaptic AP waveform at rest (10). It is unclear if mitochondrially produced ATP also fuels these pumps during activity.

In this study, we test the independent contribution of the two major modes of ATP production to support activity-dependent presynaptic function at the calyx of Held, a giant glutamatergic relay synapse in the auditory brainstem specialized for high-frequency transmission, in acute brain slices. This terminal accumulates a high concentration of synaptically localized mitochondria in the mature synapse (22, 23) and has a massive pool of SV available for release (24–26). We examined the effect of selectively and acutely impairing glycolysis or OxPhos on presynaptic function during bouts of high-frequency activity. On the whole, we observed that selective inhibition of either ATP production pathway did not affect synaptic depression or recovery during short bursts of high-frequency activity. Only when driving transmission at high frequency for extended periods, several minutes in mature animals, do we consistently observe defects in synaptic transmission. Mature synapses had no glycolytic requirement if pyruvate was present, but lost transmission if OxPhos was inhibited. During sustained high-frequency activity, APs failed first, followed by dysregulation in SV recycling and release. Modeling of synaptic failures showed ATP demand increased supralinearly with sustained stimulation.

## MATERIALS AND METHODS

### Animals

All animals in this study were used in accordance with animal welfare protocols approved by the Institutional Care and Use Committee at the University of Nevada, Reno, and in accordance with the Guide for the Care and Use of Laboratory Animals (27). C57BL/6j mice (Charles River Labs) *postnatal days 8–10* and *16–18* of both sexes were used for this study. A total of 115 animals were used.

### Preparation of Acute Brain Stem Slices

Brain stem slices were made as described previously (10). Briefly, mice were euthanized via rapid decapitation, and the brain removed from the skull and submerged in ice-cold slicing artificial cerebrospinal fluid (ACSF) solution, containing the following (in mM): 85 NaCl, 2.5 KCl, 25 glucose, 25 NaHCO_3_, 1.25 NaH_2_PO_4_, 75 sucrose, 0.5 CaCl_2_, 7 MgCl_2_, 3 myo-inositol, 2 Na-pyruvate, 0.4 ascorbic acid; pH 7.3 when bubbled with carbogen gas (95% O_2_–5% CO_2_), and density ∼310 osmol/kgH_2_O. Transverse brain stem slices containing the medial nucleus of the trapezoid body (MNTB) were made at a thickness of 200 µm using a vibratome (VT 1200S, Leica Microsystems, Oberkochen, Germany). Slices were transferred to an incubation chamber containing recording ACSF bubbled with carbogen gas for 30–60 min at 35°C and maintained thereafter (up to 4 h) at room temperature (∼23°C) until used for recording.

### Electrophysiology

Slices were transferred to a recording chamber and perfused at ∼2 mL/min with ACSF solution bubbled with carbogen gas. Recording ACSF was composed of the following (in mM): 125 NaCl, 2.5 KCl, 1 glucose, 25 NaHCO_3_, 1.25 NaH_2_PO_4_, 1.2 CaCl_2_, 1 MgCl_2_, 3 myo-inositol, 2 Na-pyruvate, 0.4 ascorbic acid, unless otherwise indicated. Sucrose (a nonmetabolizable substrate) was added to solutions to compensate for loss of glucose, and maintain bath density at 315–320 osmol/kgH_2_O. All recordings were performed at 33°C–35°C, maintained by an in-line heater (Warner Instruments, Holliston, MA). Slices were visualized using infrared gradient contrast (28) with a ×60 water-immersion objective (Olympus), and monitored with a CCD camera (QIClick, QImaging; Surrey, BC, Canada). Whole cell patch clamp recordings were made using a HEKA EPC-10/2 amplifier controlled by Patchmaster software (HEKA, Ludwigshafen/Rhein, Germany; RRID:SCR_000034). Data were low-pass filtered at 2.9 kHz and digitized at sampling rates of 10 kHz. Pipettes were pulled from thick-walled borosilicate capillary glass (1B200F-4; WPI, Sarasota, FL) using a P-1000 pipette puller (Sutter Instruments, Novato, CA; RRID:SCR_021042) to tip resistances of 1.5-3 MΩ. Postsynaptic voltage-clamp recordings from principal cells of the MNTB used a pipette solution containing (in mM) 130 cesium gluconate, 10 CsCl, 5 sodium phosphocreatine, 10 HEPES, 5 EGTA, 10 TEA-Cl, 4 Mg-ATP, 0.5 GTP, 5 QX-314 adjusted to pH of 7.2 with CsOH, and 310–315 osmol/kgH_2_O. Series resistances for voltage clamped cells ranged from 2–10 MΩ, and series resistance (R_s_) was routinely compensated to <0.5 MΩ for the duration of the recording. Cells were routinely held at −71 mV command voltage and corrected for liquid junction potential (−11 mV, from pClamp LJP calculator; RRID:SCR_011323). Excitatory postsynaptic currents (EPSCs) were evoked by placing a bipolar stimulating electrode near the midline and applying a biphasic voltage waveform (100-µs duration, <2.5 V). All evoked EPSC recordings were induced by applying stimulation ∼0.5 V over threshold. Increased voltages (up to 1 V over threshold, more than ×2 threshold) did not further facilitate AP propagation or efficacy of transmission (data not shown). AMPA-mediated EPSC recordings were performed in normal ACSF solution, with added 50 µM D-AP5 to block NMDA receptors, 0.5 µM strychnine to inhibit glycine receptors, and 10 µM bicuculline to inhibit GABA_A_ receptors. In experiments from immature mice kynurenic acid (2 mM) was included in the bath solution to block AMPA receptor desensitization (29). Salts and pharmacological agents were purchased from Sigma-Aldrich (St. Louis, MO), Tocris Biosciences/R&D Systems (Minneapolis, MN), and/or Alomone Labs (Jerusalem, Israel). Electrophysiology data were analyzed offline using prepackaged and custom-written analysis routines in IGOR Pro (Wavemetrics, Lake Oswego, OR; RRID:SCR_000325).

### Estimating Presynaptic Depression Kinetics

Paired pulse ratio was determined as peak EPSC_2_/EPSC_1_. For depression kinetics, traces were normalized and fit with a single exponential (3rd-20th pulse for 100 Hz, 3rd-46th pulse for 300 Hz). Steady state was calculated from the mean of all events at time points > 3 × τ. Successful postsynaptic events were determined visually, and threshold set per cell, usually at ∼5% of initial EPSC. Transmission successes were reported as percentage of successes on a 25-event rolling average.

### Recovery from Synaptic Depression

The readily releasable pool (RRP) was depleted with 100–300 Hz trains, and fractional recovery of the RRP and single EPSCs were measured following increasing rest periods from 20 ms–13 s between pairs of trains, ≥30 s rest intervals were allowed after each pair of trains for synaptic recovery and dissipation of cytosolic Ca^2+^ buildup. Relative RRP was measured as the current integral during the train after subtracting stimulation artifacts to capture both synchronous and asynchronous release events, as previously described (30). RRP recovery time constant was determined per cell as the result of a single exponential fit.

### Ca^2+^ Imaging

Adeno-associated virus encoding the genetically encoded Ca^2+^ sensor GCaMP6m (AV-1-PV2823, Penn Vector Core, University of Pennsylvania; 31) was injected into the ventral cochlear nucleus (VCN) at P1. After a 7-day incubation period, GCaMP6 was visible at the presynaptic calyceal nerve terminal during orthodromic stimulation. Midline stimulation (100 Hz, 500 ms) was used to visually identify infected terminals with low resting [Ca^2+^]_free_, and validated axonal connectivity at 10 Hz using an EM-CCD camera (Hamamatsu ImagEM X2; Bridgewater, NJ). Resting Ca^2+^ was imaged during drug application without additional stimulation at 1 Hz for 10–15 min, using descriptor-based image registration plugin for FIJI to correct for image drift (32). Images including a stimulated response were used to define the region of interest. Activity-dependent change in fluorescence was reported as δF/F_0_ (%), normalized to the first 10 frames (∼2 min) in the series.

### Selective Inhibition of Glycolytic versus Mitochondrial ATP Production

Inhibition of glycolysis and mitochondrial production of ATP and selectivity of iodoacetic acid and oligomycin has been described previously (10). Briefly, glycolysis was blocked by excluding glucose from the extracellular ACSF and inclusion of 1 mM iodoacetic acid (IAA), a commonly used inhibitor of glyceraldehyde dehydrogenase in brain slice preparations (33–36). We refer to this as “−glycolysis” in the results. Mitochondrial ATP synthesis was blocked by exclusion of sodium pyruvate from ACSF and inclusion of 1 μM oligomycin A, a commonly used inhibitor of the mitochondrial ATP synthase (14, 37, 38), and referred to as “−OxPhos” in the results. Importantly, use of oligomycin is specific to the ATP synthase and does not disrupt mitochondrial potential or mitochondrial Ca^2+^ buffering (39). In normal conditions and where mitochondrial respiration was blocked, glucose was reduced to 1 mM and Ca^2+^ to 1.2 mM, unless otherwise indicated, to more closely mimic conditions in vivo (40, 41). In all cases, the slices were perfused with the experimental buffer (−glycolysis or −OxPhos) for >8 min after establishing whole cell recording configuration, to allow equilibration of the bath and penetration of drug into the cells. Our previous work in this preparation suggest that this is sufficient time to inhibit the targeted ATP production pathways (10). One cell was recorded per slice after exposure to drug. In all recordings, postsynaptic ATP was maintained at physiological levels (∼4 mM) by inclusion in the recording pipette solution, as previously published (10). Thus, the changes observed are primarily, if not exclusively, due to acute effects of disrupted ATP metabolism on presynaptic function.

### Experimental Design and Statistical Analysis

Experimental groups were Control (1 mM glucose), IAA-treated (−glycolysis), oligomycin-treated (−OxPhos), and IAA + oligomycin-treated slices. Statistics were calculated using Prism 9.1.2 software (GraphPad Software, La Jolla, CA; RRID:SCR_002798). Statistical significance was determined by appropriate tests: paired *t* test, one-way ANOVA, or two-way ANOVA with post hoc correction for multiple comparisons, as indicated in results and are reported with degree of freedom and sample size effect. In most cases, differences are compared with control replicates (N) and are counted per cell recording for each condition. Significance is illustrated in figures as *P < 0.05, **P < 0.01, and ***P < 0.001. Data are presented as means ± SE.

### Modeling ATP-Dependent Failure Rate

The dynamics of energy consumption during sustained high-frequency stimulation trains were modeled by the differential equation:

(*1*) $$\frac{d\left[ATP\right]}{dt}=k-\gamma_{b}\left[ATP\right]-\gamma_{i}\left[ATP\right],$$

where [*ATP*] represents the level of ATP, *γ_b_* is the basal stimulation-independent consumption of ATP, and *γ_i_* is the consumption during the *i^th^* stimulation train. Assuming a constant ATP consumption γ_1_ during the first train, solving the above differential equation yields the following decay in ATP levels over time:

(*2*) $$\left[ATP\right]=\frac{1+\frac{\gamma_{1}}{\gamma_{b}}e^{-(\gamma_{1}+\gamma_{b})t}}{1+\frac{\gamma_{1}}{\gamma_{b}}}\;{\left[ATP\right]}_{1},$$

where ${\left[ATP\right]}_{1}=\frac{k}{\gamma_{b}}$ is the basal ATP level at the start of the first sustained stimulation train, essentially at rest. The failure rate is modeled as a sigmoidal Hill function of the ATP level:

(*3*) $$Failure\;Rate=\;\frac{1}{1+{\left(\frac{\left[ATP\right]}{K}\right)}^{h}}\;\times 100\text{\%},$$

which increases with decaying energy levels over time. Here *h* denotes the Hill coefficient and *K* is the ATP level at which the failure rate is 50%. Substituting *Eq. 2* in *Eq. 3* predicts the increasing failure rate over time.

To fit *Eq. 3* to data, we determine the OxPhos-dependent failure rate by taking the difference of the success rate between 1 mM glucose with and without oligomycin. We estimate the model parameters by performing a least-square fitting using the Solver toolbox in Microsoft Excel with the Generalized Reduced Gradient Nonlinear method (RRID:SCR_016137).

Our analysis determined values for cooperativity between ATP and successful transmission (*h*), basal and activity-dependent ATP consumption (*γ_b_*,γ_1_), and relative energy increase ($\frac{\gamma_{1}}{\gamma_{b}}$) between stimulated and basal conditions, for the first train. Moreover, *K*/[*ATP*]_1_ can be used to estimate the relative ATP level when failure rate reaches 50%. Finally, we point out that a constant consumption rate, γ_1_, is sufficient to provide a good fit to the experimentally measured increase in failures during the first stimulation train.

For the second stimulation train, the failure rate becomes higher than during the first train, suggesting an increased ATP demand. To account for this, we consider a dynamic ATP consumption rate for the second train that is phenomenologically modeled as an increasing sigmoidal over time

(*4*) $$\gamma_{2}\left(t\right)=\gamma_{1}+(\gamma_{2}^{max}-\gamma_{1})\frac{t^{h_{2}}}{K_{2}^{h_{2}}+t^{h_{2}}}.$$

We assume at the start of the second train (*t* = 0), the consumption rate is the same as that in the first train γ_2_(0) = γ_1_, and over time the consumption rate increases to $\gamma_{2}^{max}$ with parameters *h*_2_ and *K*_2_ defining the sigmoidal. Given the recovery of ATP levels between the first and second stimulation trains, we assume that the ATP level at the start of the second stimulation train to be an unknown [*ATP*]_2_ that will be determined from fitting data. We numerically solve *Eq. 1* with a time-varying ATP consumption rate *Eq. 4* to predict ATP levels over time, which then yields the failure rate from *Eq. 3*. Fitting the model prediction to the measured failure rate for the second train, we determine the optimal γ_2_(*t*) and [*ATP*]_2_ that is needed to capture the increasing failures. For the third stimulation train, we again assume a time-dependent consumption rate

(*5*) $$\gamma_{3}\left(t\right)=\gamma_{1}+(\gamma_{3}^{max}-\gamma_{1})\frac{t^{h_{3}}}{K_{3}^{h_{3}}+t^{h_{3}}},$$

with parameters $\gamma_{3}^{max}$, *h*_3_, $K_{3}^{h_{3}}$ to be determined from fitting data. We stress that this model was derived to match the experimental data, with no biological constraints, and few assumptions.

## RESULTS

Persistent synaptic activity leads to presynaptic depression at the calyx due to SV depletion of the readily releasable pool (RRP; 42), which stimulates ATP- and Ca^2+^-dependent pool refilling (43, 44). This study aimed to determine the role of glycolysis versus mitochondrial OxPhos to supply energy for sustained presynaptic transmission and whether these pathways exhibit changes during synaptic maturation after the onset of hearing, at the mouse calyx of Held. Previous work has shown that blocking both pathways results in loss of transmission within minutes, even in the absence of stimulation (10, 17, 45). As a result, we inhibited glycolysis or mitochondrial respiration selectively in a majority of the experiments described below. In addition, most previous slice work at the calyx of Held synapse has used 10–25 mM glucose, hyperglycemic relative to in vivo glucose concentration (1–2.5 mM; 46). We investigated transmission efficacy at near physiological conditions: 1 mM glucose, 1.2 mM Ca^2+^, and 33–35°C, similar to conditions used in previous work at this same synapse (17).

### ATP Derived from Both Glycolysis and Mitochondrial OxPhos Support High-Frequency Transmission at the Developmentally Immature Calyx

The energetic basis for RRP refilling during ongoing synaptic activity at the immature calyx (8–10 days after birth) was tested by selectively impairing glycolysis or OxPhos, as described in materials and methods. The afferent fiber was stimulated with a high-frequency train (HFS_100_: 100 Hz, 200 ms) sufficient to induce depression and deplete the RRP. AMPAergic responses were recorded from the innervated MNTB principal cell. In these recordings from immature animals, 2 mM kynurenic acid was used to avoid receptor desensitization (materials and methods). Because ATP production is tightly regulated by activity (2, 5) and because no effect of oligomycin was observed at rest (10), we conditioned cells with multiple HFS_100_ trains (9 pairs of HFS_100_ trains over the course of 5 min, with varying rest intervals, using the Recovery from Synaptic Depression Protocol, see materials and methods) to drive activity and increase ATP demand. This protocol was repeated four times (“Runs”) over the course of 20 min, in total. Similar to our previous report (10), there was no difference in average EPSC size at rest due to blockade of glycolysis or OxPhos under these recording conditions [*P* = 0.312; *F*(2, 30) = 1.210; *n* = 9–16 cells per condition; one-way ANOVA].

**Figure 1.**
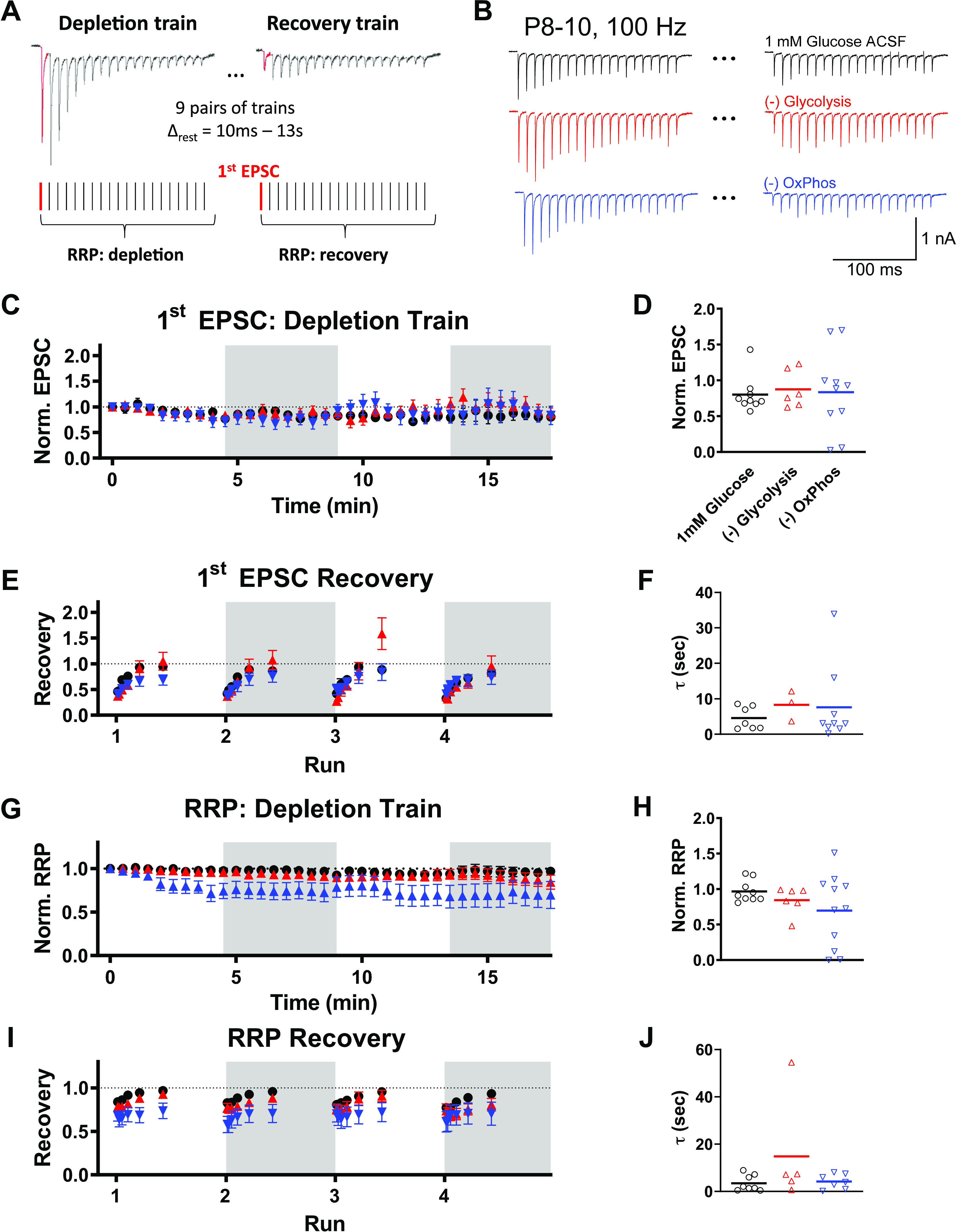
Recovery from synaptic depression in immature synaptic terminals. *A*: illustration of recovery from depression protocol. Pairs of HFS_100_ trains were delivered with increasing intervals between trains, from 10 ms to 13 s. Rest intervals of 30 s was given between pairs of trains to allow adequate recovery of the releasable pool in control conditions. First EPSC amplitude was compared between the depression train and recovery train (red traces). Alternatively, the integral of the responses during the train was used to approximate readily releasable pool size and relative recovery (red plus black traces). Four sets of these trains were given over the course of ∼20 min. *B*: example pairs of depression and recovery traces. Shown are depression and recovery traces with 400-ms interstimulus interval, during the first run. *C*: EPSC amplitude of the first response in every depletion train, normalized to the initial EPSC amplitude. Symbols are mean of recordings, per condition. Open and shaded sections correspond to different “runs,” 1 mM glucose (black, *n* = 9 cells), −glycolysis (red, *n* = 6 or 7 cells), and −OxPhos conditions (blue, *n* = 10–15 cells) are shown. Variability in replicates are due to attenuation of viable recordings. On average, initial EPSC amplitudes at the start of the depletion trains were not altered even at the end of the four runs [*P* = 0.9815, *F*(3, 26) = 0.05743; one-way ANOVA]. *D*: comparison of EPSC amplitude between treatment conditions at the end of the fourth run (20 min of repetitive stimuli). Symbols represent individual recordings. *E*: recovery time course of first EPSC, shown for each run. *F*: comparisons of recovery time constant (τ) of the fourth run, shown for single cells. Recordings that could not be adequately fit were excluded (1 mM glucose *n* = 7, −glycolysis *n* = 3, −OxPhos *n* = 9). *P* = 0.6427, *F*(3, 23) = 0.5663; one-way ANOVA. *G*: integral of responses of HFS_100_ depression train, used as an estimate of RRP size, normalized to the RRP size from the first depletion train. *H*: comparison of RRP size after ∼20 min stimulation. RRP size was not significantly different between conditions. *P* = 0.1872, *F*(2.000, 15.36) = 1.872; ANOVA with Brown–Forsythe correction for unequal SD. *I*: time course of RRP recovery, shown for each run. *J*: comparisons of RRP recovery time constant (τ) of the fourth run, shown for single cells. Points before 400 ms were not used for fits, because RRP facilitated before recovery in 1.2 Ca. Recordings that could not be adequately fit were excluded (1 mM glucose *n* = 8, −glycolysis *n* = 5, −OxPhos *n* = 7) . Recovery kinetics were not altered by blocking either glycolysis or OxPhos, relative to recordings in 1 mM glucose [*P* = 0.2471, *F*(3, 21) = 1.486; one-way ANOVA]. EPSC, excitatory postsynaptic current; HFS_100_, high-frequency stimulation train; Norm., normalized; RRP, readily releasable pool.

Using pairs of HFS_100_ trains at intervals from 10 ms to 13 s permits measurement of recovery after RRP depletion (illustrated in Fig. 1, *A* and *B*). Using this protocol, we measured single EPSCs at the beginning of the depletion trains, given at 30 s rest intervals, across each Run (Fig. 1, *C* and *D*). The first EPSC amplitude at the beginning of each depletion train was not affected by activity, nor by blockade of either glycolysis or OxPhos (Fig. 1*C*). On average, initial EPSC amplitudes at the start of the depletion trains were not altered even at the end of the four Runs [*P* = 0.9815, *F*(3, 26) = 0.05743; one-way ANOVA]; however, the relative first EPSC amplitude at the final stimulation train did show increased variability when OxPhos was blocked (Fig. 1*D*). Coefficient of variation (CV) for these responses after 17.5 min of bursting activity (and 27 min exposure) was more than doubled by blocking OxPhos (1 glucose = 0.31, −glycolysis = 0.30, −OxPhos = 0.68). The first EPSC in the recovery train was also examined as a measure of functional transmission recovery (Fig. 1, *E* and *F*). The time course of recovery of a single EPSC during each run was fit by a single exponential (Fig. 1*E*). Recovery time constants at the end of the protocol (Run 4, 17.5 min activity) were not affected by blocking glycolysis or OxPhos (Fig. 1*F*). Due to variability in recovery of individual recordings, some cells could not be adequately fit (1 mM glucose *n* = 2/9, −glycolysis *n* = 3/6, −OxPhos *n* = 2/11 were not fit).

Estimation of readily releasable pool (RRP) size and recovery were also retrieved from this protocol, using the integral of responses during each depletion train, as described previously (30). RRP size was not altered by activity in 1 mM glucose or by loss of glycolysis and was marginally decreased when OxPhos was blocked (Fig. 1*G*). At the end of the protocol, RRP size was not significantly different between conditions (Fig. 1*H*). Similar to evaluation of the first EPSC during the depletion train, the RRP CV more than tripled when OxPhos was blocked (1 glucose = 0.16, −glycolysis = 0.23, −OxPhos = 0.73). Kinetics of RRP recovery during each run were also compared and fit with a single exponential in a majority of recordings (Fig. 1*I*). Recovery kinetics were not altered by blocking either glycolysis or OxPhos, relative to recordings in 1 mM glucose (Fig. 1*J*). Similar to EPSC recovery time course, cells that could not be fit were excluded (1 mM glucose *n* = 1/9, −glycolysis *n* = 1/6, −OxPhos *n* = 4/11 were not fit).

**Figure 2.**
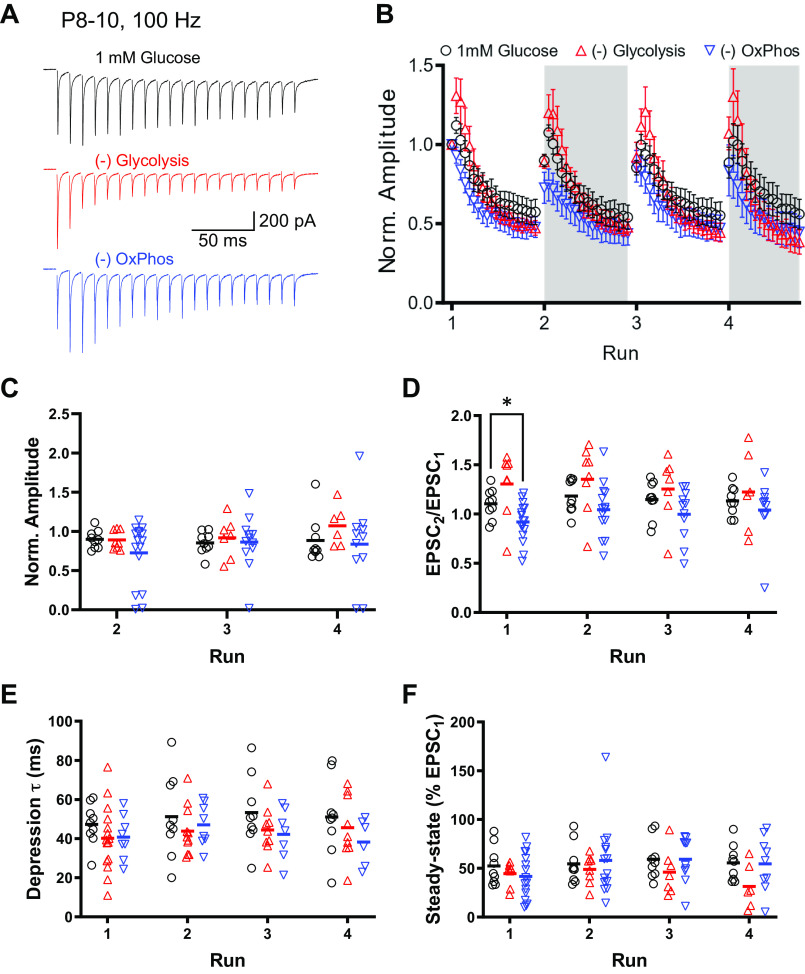
Short duration stimulation trains in immature calyx of Held terminals. Depletion trains (100 Hz, 200 ms; HFS_100_) were delivered repetitively over 20 min of recording. The effect of selectively blocking glycolysis (red traces) or OxPhos (blue traces) were compared with physiological glucose (1 mM). Each train shown is the average of nine HFS_100_ “depletion” trains per run, with 30 s rest between train pairs. *A*: representative depletion trains. shown are the average of HFS_100_ responses for depletion trains during the fourth “run.” *B*: EPSC peaks during HFS_100_ “depletion” trains for each run, normalized to the first HFS_100_ depletion train. Symbols are mean events per condition. Replicates are the same as Fig. 1: 1 mM glucose (black, *n* = 9 cells), −glycolysis (red, *n* = 6 or 7 cells), and −OxPhos conditions (blue, *n* = 10–15 cells). *C*: first EPSC amplitude during HFS_100_ shown for each run, normalized to response amplitude before stimulation train. Symbols represent amplitudes from individual recordings. EPSC amplitude at the beginning of each initial depression train was not affected by either run number [*P* = 0.0565, *F*(2.307, 59.97) = 2.881] or energetic blockade [*P* = 0.3408, *F*(2, 30) = 1.116; two-way ANOVA]. *D*:paired-pulse ratio of first two responses in HFS_100_, shown per run. Paired-pulse ratio was not significantly altered by run number [*P* = 0.1226, *F*(2.474, 64.33) = 2.481; two-way ANOVA]. There was a small decrease in paired-pulse ratio when OxPhos was blocked in the first HFS_100_ run [*P* = 0.0290 vs. 1 glucose, *F*(2, 30) = 4.688, two-way ANOVA followed by multiple comparison]. *E*: depression time course was fit with a single exponential. Time constant (τ) is shown for HFS_100_ per run. No differences were present either across run number [*P* = 0.4310, *F*(2.504, 56.77) = 0.9018], or treatment condition [*P* = 0.1864, *F*(2, 29) = 1.781; two-way ANOVA]. *F*: steady-state depression during HFS_100_, plotted as percentage of EPSC amplitude before stimulation train, shown per run. Blocking either glycolysis or OxPhos selectively had no effect on steady-state responses [time *P* = 0.1064, *F*(2.279, 54.71) = 2.268; treatment *P* = 0.5086, *F*(2, 30) = 0.6916; two-way ANOVA]. EPSC, excitatory postsynaptic current; HFS_100_, high-frequency stimulation train; Norm., normalized; OxPhos, oxidative phosphorylation.

We observed very minor changes in short-term plasticity during the HFS_100_ depletion trains due to blockade of either glycolysis or OxPhos, from the same recordings (Fig. 2). We examined the short-term depression characteristics of the depletion trains, averaged per run for each cell (Fig. 2*B*). On average, EPSC amplitude at the beginning of each initial depression train was not affected by either run number or energetic blockade. However, cells in the −OxPhos condition showed greater variability, with two cells losing transmission during the second run, and other cells elevating EPSC amplitude in the third and fourth runs (Fig. 2*C*). This increased variability is consistent with previous reports where energetic capacity is impaired (10, 17). Paired-pulse ratio was not significantly altered by run number. There was a small decrease in paired-pulse ratio when OxPhos was blocked in the first HFS_100_ run, relative to 1 mM glucose, but was not evident in subsequent runs (Fig. 2*D*). Cells that lost responses were not included in this or subsequent analyses. We compared the time course of depression within these trains, fit with a single exponential decay function, to evaluate whether blocking glycolysis or OxPhos affects short-term plasticity (Fig. 2*E*). No differences were present either across run number, or treatment condition. Similarly, steady-state responses were evaluated, which represent a balance of SV depletion due to release and activity-dependent refilling of release sites (Fig. 2*F*). Again, blocking either glycolysis or OxPhos selectively had no effect on steady-state responses.

**Figure 3.**
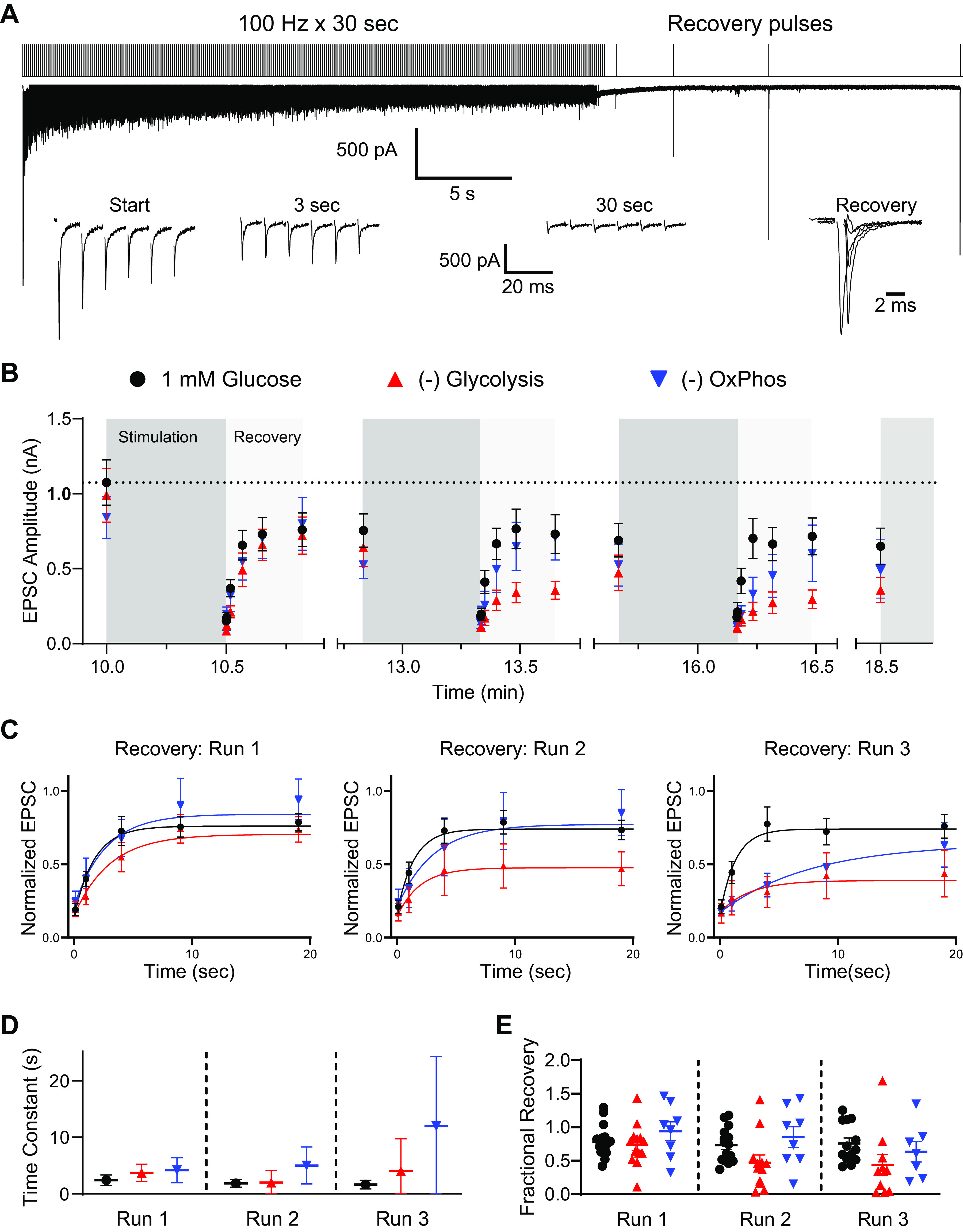
Depression and recovery due to sustained high-frequency trains in immature synaptic terminals. Sustained high-frequency stimulation (100 Hz, 30 s) was delivered, followed by single test pulses at 0.1, 1, 4, 9, and 19 s after the end of the stimulation train. *A*: stimulation protocol (*top*) and example recording (*middle*) are shown. *Insets* at *bottom* show excerpts of responses during the stimulation train illustrating synaptic depression, and recovery responses from the sample recording (in 1 mM glucose). *B*: EPSC amplitudes at the beginning and end of sustained stimulation train and responses to test pulses map depression and recovery. Symbols represent mean of recordings, per condition (1m glucose *n* = 13–16, −glycolysis *n* = 10–13, −OxPhos *n* = 7 or 8). This protocol was given three times during each recording, with 2 min rest between depression and recovery protocol (not shown). Depression relative to initial EPSC amplitude was similar across all runs in 1 mM glucose, and where glycolysis or OxPhos were blocked [*P* = 0.3934, *F*(11, 167) = 1.064; one-way ANOVA]. *C*: time course of recovery, normalized to EPSC before sustained stimulation train, were fit with a single exponential for average of recovery responses. Symbols are mean of recordings, per condition. Recovery following the three subsequent stimulation trains are shown. *D*: comparison of time constant of recovery (τ), shown following each sustained stimulation train. Symbols are mean of recordings per condition. Errors represent asymptotic means ± SE of time constant fit. Time constants for recovery were not significantly affected by blocking glycolysis or OxPhos, relative to 1 mM glucose [*P* = 0.7213; *F*(8, 93) = 0.6645; one-way ANOVA]. *E*: fractional recovery of individual cells at 19 s after end of each sustained stimulation train. Recovery was decreased when glycolysis was blocked, especially after the second and third trains, but not when OxPhos was blocked [*P* = 0.0546, *F*(2, 32) = 3.188, and *P* = 0.1784, *F*(2, 27) = 1.839, respectively; one-way ANOVA]. EPSC, excitatory postsynaptic current; OxPhos, oxidative phosphorylation.

In an attempt to further stress presynaptic function and drive ATP consumption, we drove transmission using sustained stimulation trains (30 s at 100 Hz), and followed synaptic recovery in these immature synapses (Fig. 3). The stimulation protocol is illustrated in Fig. 3*A*. Synaptic transmission was largely maintained after a sharp initial depression in EPSC size, though a second slower form of depression emerged. Synaptic recovery was monitored with single test pulses over the subsequent 20 s, followed by a 2-min rest interval. This protocol was repeated three times (Fig. 3*B*). Depression relative to initial EPSC amplitude was similar across all runs in 1 mM glucose and where glycolysis or OxPhos were blocked. Transmission recovery was measured as EPSC amplitude with single test pulses and showed progressive loss of recovery when glycolysis was blocked, relative to 1 mM glucose (Fig. 3*C*). Due to variability in responses per cell, the average time constant for recovery was measured as a single exponential, and asymptotic errors presented in Fig. 3*D*. Time constants for recovery were not significantly affected by blocking glycolysis or OxPhos, relative to 1 mM glucose, though confidence in the recovery time constant was reduced following the third depression train (error bars in Fig. 3*D*).

Fractional recovery was also measured at the end of the 20-s recovery period following the depression train (Fig. 3*E*). Recovery was slightly decreased when glycolysis was blocked, especially after the second and third trains, but not when OxPhos was blocked (Fig. 3*E*). These results suggest that both glycolysis and OxPhos are required for full recovery following sustained stimulation, and may be acting through separable mechanisms.

**Figure 4.**
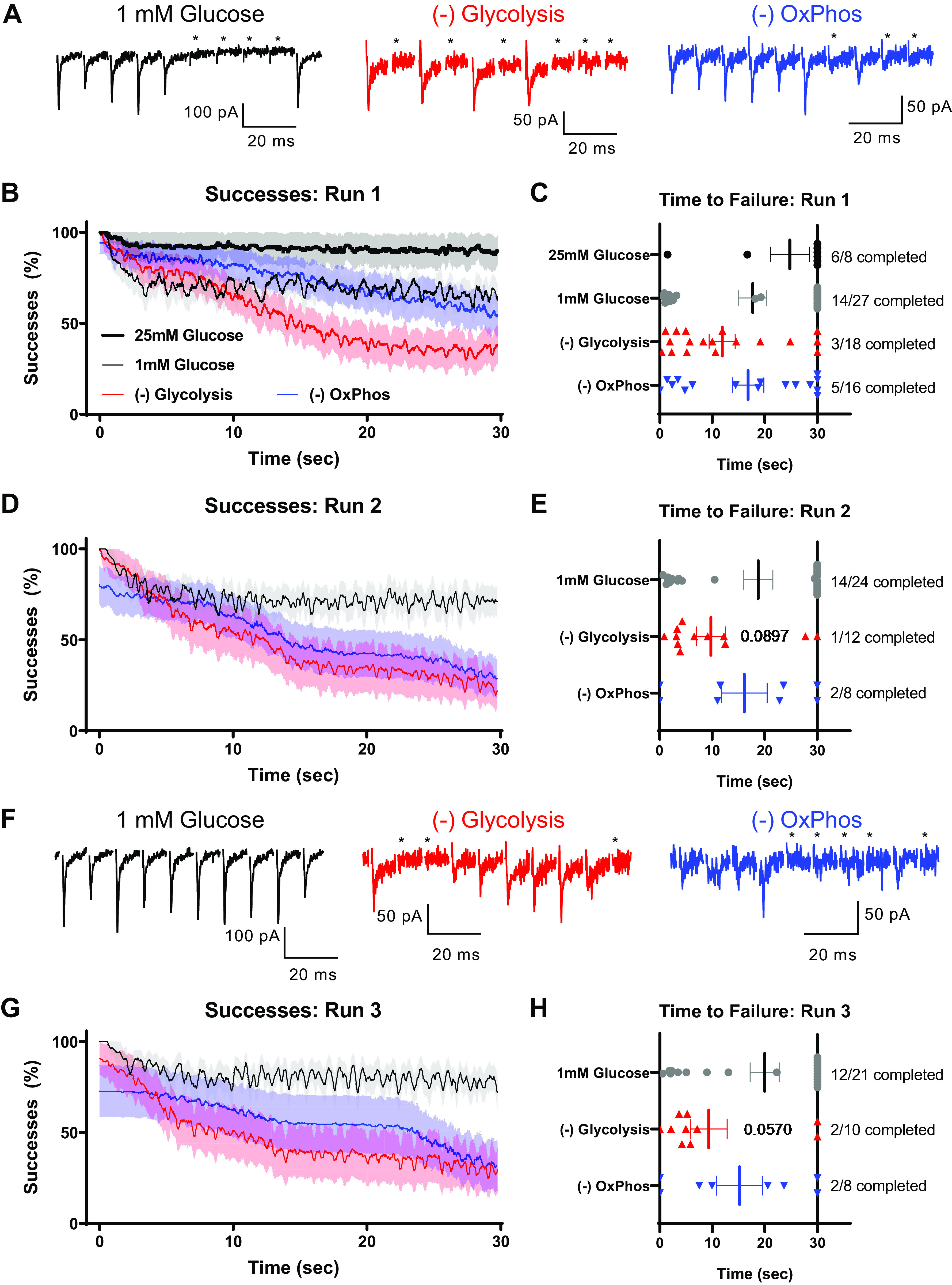
Transmission failures occur during sustained stimulation in immature synaptic terminals. Successes were recorded as events that showed a clear EPSC response relative to baseline. In all conditions, some occasional failures were observed. These data are from the same recordings as Fig. 3. *A*: example recordings from the end of the first sustained stimulation train. Failures are marked with asterisks. *B*: successful responses plotted during sustained stimulation train, as running average of successes per 25 stimuli. Note inclusion of 25 mM glucose (heavy black line) as a control, in addition to 1 mM glucose (light black line). *C*: time to first failure per cell for the first sustained stimulation train. “Failure” was defined as two subsequent stimuli without a corresponding postsynaptic EPSC. Symbols are results of individual recordings. Numbers to right of plot illustrate fraction of recordings that showed no subsequent failures. *D*: successful responses plotted during the second sustained stimulation train, as running average of successes per 25 stimuli. *E*: time to first failure per cell for the second sustained stimulation train. Numbers to right of plot illustrate fraction of recordings that showed no subsequent failures. *P* value refers to mean time-to-failure (one-way ANOVA). *F*: example recordings from the end of the third sustained stimulation train. Failures are marked with asterisks. *G*: successful responses plotted during third sustained stimulation train, as running average of successes per 25 stimuli. *H*: time to first failure per cell for the third sustained stimulation train. Numbers to right of plot illustrate fraction of recordings that showed no subsequent failures. *P* value refers to mean time-to-failure (one-way ANOVA). EPSC, excitatory postsynaptic current; OxPhos, oxidative phosphorylation.

We occasionally saw transmission failures during these sustained high-frequency trains: a complete lack of response embedded in a train of EPSCs. We infer that these were failures due to intermittent loss of AP propagation, since depression before transmission failures was not to the level of single quanta, and subsequent successful responses were seen. Examples of these events are illustrated in Fig. 4*A*, at the end of the first train of stimuli. We carefully evaluated these transmission failures independently of response amplitude, in an attempt to deconvolve transmission failures due to AP failure and loss of available SVs. Transmission successes were plotted as a running average of 25 events during the depression train (Fig. 4*B*), and the time to failure was determined as the first occurrence of two subsequent stimulations lacking corresponding EPSCs (Fig. 4*C*). Loss of glycolysis, but not OxPhos, resulted in increased failures during the train, and earlier time to failure. Subsequent stimulation trains did not result in increased number of failures in 1 mM glucose, but resulted in progressively more failures when either glycolysis or OxPhos were blocked (Fig. 4, *D*–*H*). These results suggest that both glycolysis and OxPhos are required to support high-frequency transmission at the immature calyx of Held synapse.

Interestingly, we observed a number of failures in 1 mM glucose at this age, far more than in “standard” recording conditions (25 mM glucose), indicating energy supply may be limiting for high-frequency transmission in these immature synapses. Activity-dependent glycolytic production of ATP is tightly regulated by phosphofructokinase and pyruvate kinase (47, 48), but our results suggest additional bath glucose may be a caveat when examining neuroenergetics. Synaptic transmission failures are also observed in in vivo recordings from MNTB in young mice at this age, at a similar failure rate (∼20%; 49), validating the use of 1 mM glucose as an approach to mimic physiological conditions in slice recordings, to more closely mimic glucose supply in vivo (40, 41).

### Sustained High-Frequency Transmission Is Principally Supported by OxPhos at the Mature Calyx of Held

Using approaches similar to those in Figs. 1 and 2, we investigated whether the utilization of glycolysis or OxPhos shifted following postnatal maturation of the calyx of Held synapse after hearing onset. Recording from *P16–18* mice, where the calyx is functionally mature (49, 50), we drove activity using pairs of short bursts of high-frequency stimulation (HFS_300_; 300 Hz × 150 ms), separated by varying recovery intervals. This higher frequency (300 Hz vs. 100 Hz in *P8–10*) was used to induce comparable depression in these older animals as that seen in slices from younger animals (∼50% in 1 mM glucose). Nine pairs of HFS_300_ were delivered over the course of 5 min, repeated three times (four “runs”) over a 20-min recording period.

**Figure 5.**
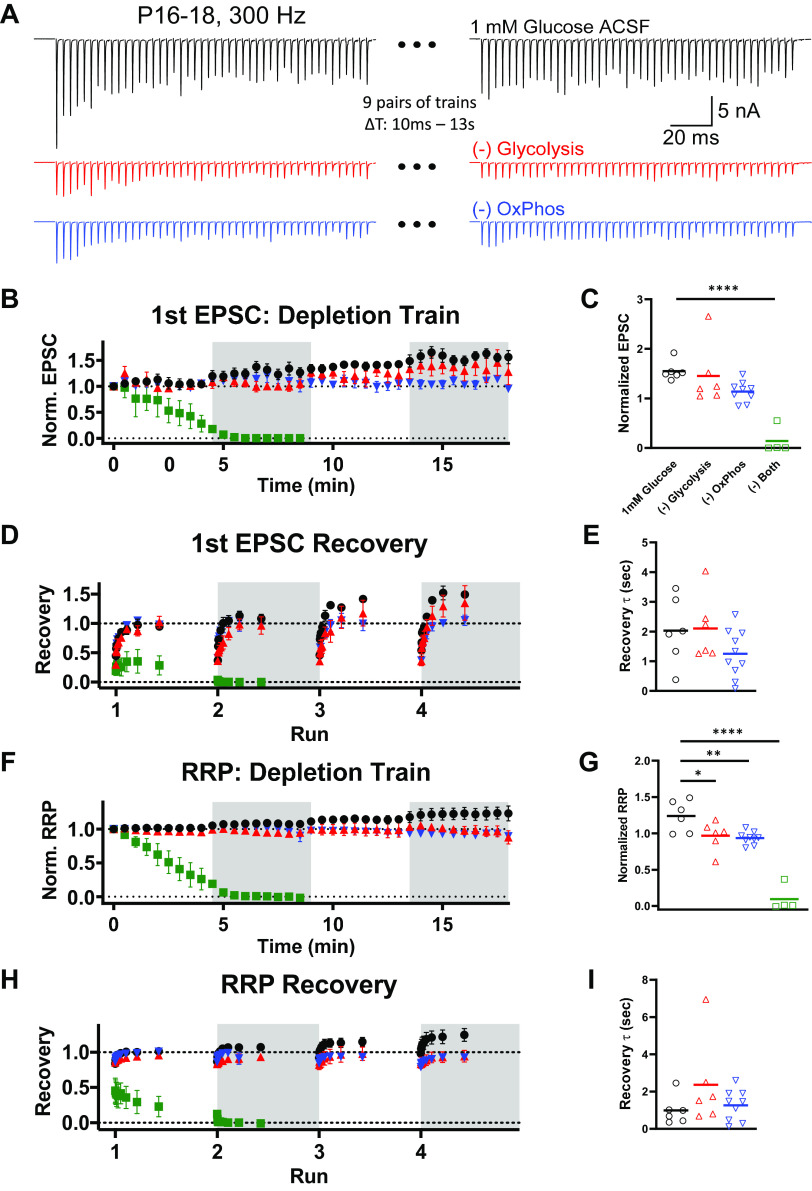
Recovery from synaptic depression after hearing onset. Pairs of HFS_300_ trains were delivered with increasing intervals between trains from 10 ms to 13 s. Rest intervals of thirty seconds was given between pairs of trains to allow adequate recovery of the releasable pool in control conditions. First EPSC amplitude and readily releasable pool (RRP) were compared between the depression and recovery trains. Four sets of these trains were given over the course of ∼20 min. *A*: example pairs of depression and recovery traces. Shown are depression and recovery traces with 400-ms interstimulus interval, during the first run. *B*: EPSC amplitude of the first response in every depletion train, normalized to the initial EPSC amplitude. Symbols are mean of recordings, per condition. Open and shaded sections correspond to different “runs,” and 1 mM glucose (black, *n* = 6–8), −glycolysis (red, *n* = 6−11), and −OxPhos conditions (blue, *n* = 10−13), as well as condition where both glycolysis and OxPhos are blocked (green, *n* = 2–4) are shown. Variability in replicates are due to attenuation of viable recordings. *C*: comparison of EPSC amplitude between treatment conditions at the end of the fourth run (20 min of repetitive stimuli). Symbols represent individual recordings. *D*: recovery time course of first EPSC, shown for each run. *E*: comparisons of recovery time constant (τ) of the fourth run, shown for single cells. Recovery was not affected by blocking glycolysis or OxPhos [*P* = 0.1736, *F*(2, 19) = 1.923; one-way ANOVA]. *F*: integral of responses of HFS_100_ depression train, used as an estimate of RRP size, normalized to the RRP size from the first depletion train. *G*: comparison of RRP size during the fourth run. Blocking glycolysis significantly limited potentiation of the RRP (*P* = 0.0223), as did blocking OxPhos (*P* = 0.0043), or both routes [*P* ≤ 0.0001, *F*(4, 27) = 35.09; one-way ANOVA]. *H*. time course of RRP recovery, shown for each run. *I*: comparisons of RRP recovery time constant (τ) of the fourth run, shown for single cells. Recovery of the RRP was not altered by repetitive stimulation, or blocking glycolysis or OxPhos selectively [*P* = 0.2226, *F*(2, 18) = 1.635; one-way ANOVA]. EPSC, excitatory postsynaptic current; HFS_300_, high-frequency stimulation train; Norm., normalized; OxPhos, oxidative phosphorylation.

Pairs of HFS_300_ trains were analyzed for single EPSC and RRP recovery following RRP depletion, using intervals ranging from 10 ms to 13 s (Fig. 5). In 1 mM glucose, EPSCs were increased due to repetitive HFS_300_ trains (Fig. 5, *A*–*C*). When glycolysis or OxPhos were blocked, this potentiation was reduced. Blocking new ATP production with oligomycin and iodoacetic acid quickly resulted in a loss of transmission during the first run (Fig. 5, *B* and *C*). Time course of recovery following the depletion train were tracked (Fig. 5*D*). Recovery of transmission was not affected by blocking glycolysis or OxPhos, even after four runs (17.5 min) of repetitive stimulation (Fig. 5*E*).

RRP recovery was also examined using this protocol. Consistent with EPSC potentiation, repetitive HFS_300_ trains increased RRP size in 1 mM glucose (Fig. 5, *F* and *G*). Blocking glycolysis significantly limited this potentiation, as did blocking OxPhos. Recovery of the RRP was not altered by repetitive stimulation, or blocking glycolysis or OxPhos selectively (Fig. 5, *H* and *I*). Taken together, these results suggest that individual responses can be maintained by either glycolysis or OxPhos; however, synaptic potentiation of EPSC and RRP requires that both routes are intact.

**Figure 6.**
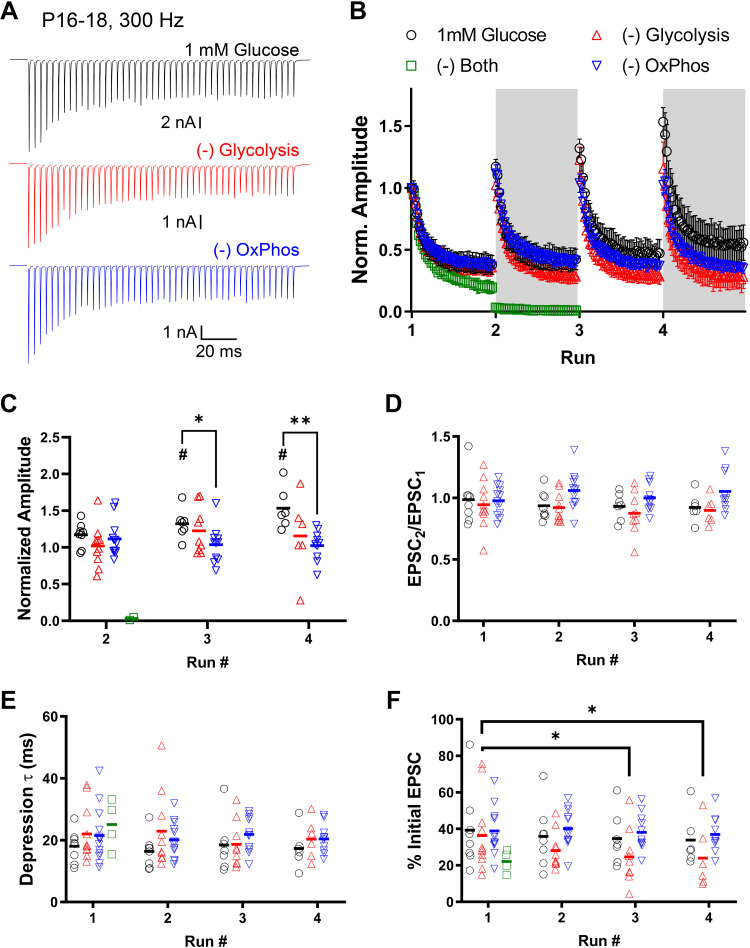
Short duration stimulation trains in calyx of Held synaptic terminals after hearing onset. Brief stimulation trains (300 Hz, 150 ms; HFS_300_) were delivered repetitively over 20 min of recording. The effect of selectively blocking glycolysis (red traces) or OxPhos (blue traces) were compared with physiological glucose (1 mM). Each “run” was composed of nine pairs of HFS_300_ stimulation, with 30 s rest between train pairs. *A*: representative depletion trains. Shown are the average of responses during HFS_300_ “depletion” trains of the fourth “run.” *B*: EPSC peaks during HFS_300_ “depletion” train of each run, normalized to the first HFS_100_ train. Symbols are mean events per condition, generated as the average of nine HFS_300_ “depletion” trains per run, with 30 s rest between train pairs. Note inclusion of condition where both glycolysis and OxPhos are blocked (green squares) and quickly results in loss of transmission. *C*: first EPSC amplitude during HFS_300_ shown for each run, normalized to response amplitude before stimulation train. Symbols represent amplitudes from individual recordings. Initial EPSC amplitude potentiated across subsequent runs, specifically for 1mM glucose [#*P* = 0.0011, *F*(2.578, 60.16) = 6.621; two-way ANOVA with post hoc comparison across runs]. Blocking OxPhos significantly decreased the initial EPSC in the third and fourth run [*, **P = 0.0328, *F*(2, 27) = 3.887; two-way ANOVA with post hoc comparison across runs]. *D*: paired-pulse ratio of first two responses in HFS_300_, shown per run. Paired-pulse ratio was not affected by run number [*P* = 0.2487, *F*(2.526, 58.94) = 1.420] or treatment [*P* = 0.1247, *F*(2, 29) = 2.239; two-way ANOVA]. *E*: depression time course was fit with a single exponential. Time constant (τ) is shown for HFS_300_ per run. Depression time constants were similar across runs [*P* = 0.4418, *F*(2.000, 46.68) = 0.8315] and were not affected by blocking either glycolysis or OxPhos [*P* = 0.3243, *F*(2, 29) = 1.171; two-way ANOVA]. *F*: steady-state depression during HFS_300_, plotted as percentage of EPSC amplitude prior to stimulation train, shown per run. Steady-state transmission was not affected in 1 mM glucose or by blocking OxPhos over multiple runs, but was affected in the third and fourth run when glycolysis was blocked [*P = 0.0034, *F*(1.549, 36.15) = 7.684; two-way ANOVA]. Treatment conditions *per se* had no effect on transmission [*P* = 0.3036, *F*(2, 29) = 1.242; two-way ANOVA]. EPSC, excitatory postsynaptic current; HFS_300_, high-frequency stimulation train; Norm., normalized; OxPhos, oxidative phosphorylation.

These brief trains of stimulation, delivered repetitively, showed no major effect when glycolysis or OxPhos were individually blocked (Fig. 6). Consistent with our previous report (10), we saw no effect of reducing glucose from 25 mM to 1 mM, blocking glycolysis, or blocking OxPhos on basal EPSC amplitude [*P* = 0.1696, *F*(3, 35) = 1.777; *n* = 7–13 cells per condition; one-way ANOVA; data not shown]. Comparing transmission between HFS_300_ runs showed potentiation of the initial EPSC amplitude across subsequent runs, specifically for 1 mM glucose (Fig. 6, *B* and *C*). Blocking OxPhos significantly decreased the initial EPSC in the third and fourth run. Blocking glycolysis had no effect on transmission. When both pathways were inhibited, transmission was lost within the first Run of HFS_300_ stimulation, and was not included in subsequent analysis (Fig. 6, *B* and *C*). Paired-pulse ratio was not affected by run number or treatment (Fig. 6*D*). EPSC amplitudes during HFS_300_ trains were fit with a single exponential to evaluate depression time course (Fig. 6*E*). Depression time constants were similar across runs and were not affected by blocking either glycolysis or OxPhos. Steady-state transmission at the end of the HFS_300_ trains was not affected in 1 mM glucose or by blocking OxPhos over multiple runs, but was affected in the third and fourth run when glycolysis was blocked (Fig. 6*F*). Treatment conditions per se had no effect on transmission.

**Figure 7.**
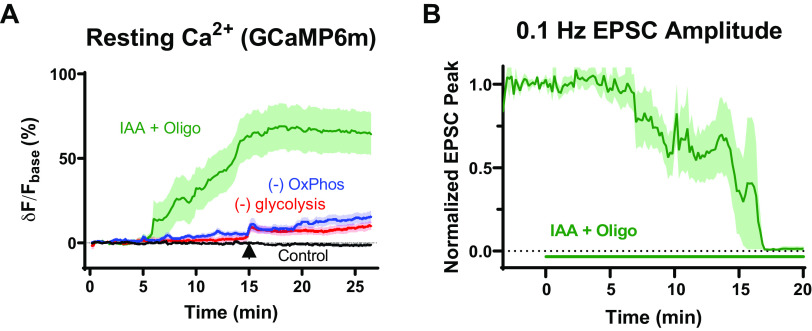
Blocking both OxPhos and glycolysis rapidly increases cytosolic Ca^2+^ and inhibits activity in mature calyx of Held terminals. Negative control recordings were made where both glycolysis and OxPhos were pharmacologically blocked. Bath solution had 1 mM iodoacetic acid (IAA), 1 μM oligomycin, zero glucose, and zero pyruvate (IAA + oligomycin condition). *A*: resting Ca^2+^ signal in calyx of Held terminals is not affected by selective inhibition of glycolysis or OxPhos. AAV-GCaMP6m was expressed in the calyx of Held terminal, and acute slices recorded at *P16–18*, under physiological conditions (33°C–35°C, 1.2 mM [Ca^2+^], 1 mM glucose in bath). Change in GCaMP6m signal was measured in calyx terminal relative to baseline. Arrow in plot corresponds to brief test pulses (100 Hz, 500 ms) to confirm region of interest and correct for lateral and focal drift. (Control *n* = 5; −glycolysis *n* = 8; −OxPhos *n* = 16; IAA + Oligo *n* = 8). Means ± SE are shown. *B*: postsynaptic responses in recordings at low-frequency stimulation (0.1 Hz). Transmission began to fail at ∼8 min after exposure to IAA + Oligomycin condition. Green bar at bottom of plot indicates addition of IAA + Oligomycin to bath. Shown is means ± SE of EPSC amplitude value from *n* = 6–8 recordings. Variability in replicate number is due to attenuated viability of recordings. EPSC, excitatory postsynaptic current; OxPhos, oxidative phosphorylation.

As a negative control to confirm that our reagents were working and that transmission is indeed affected when all net new ATP production is blocked, we monitored EPSCs due to low-frequency transmission, as well as tracking cytosolic Ca^2+^ at the calyx terminal, when both glycolysis and OxPhos were simultaneously blocked (Fig. 7). Blockade of both pathways resulted in rapid (∼5 min) increases in cytosolic Ca^2+^, which were not observed when either glycolysis or OxPhos were individually blocked (Fig. 7*A*). Transmission was quickly impaired after ∼15 min at near-physiological temperature (Fig. 7*B*). These control results confirm that our pharmacological reagents were working, and that the terminal indeed does need to continuously produce ATP to maintain function.

**Figure 8.**
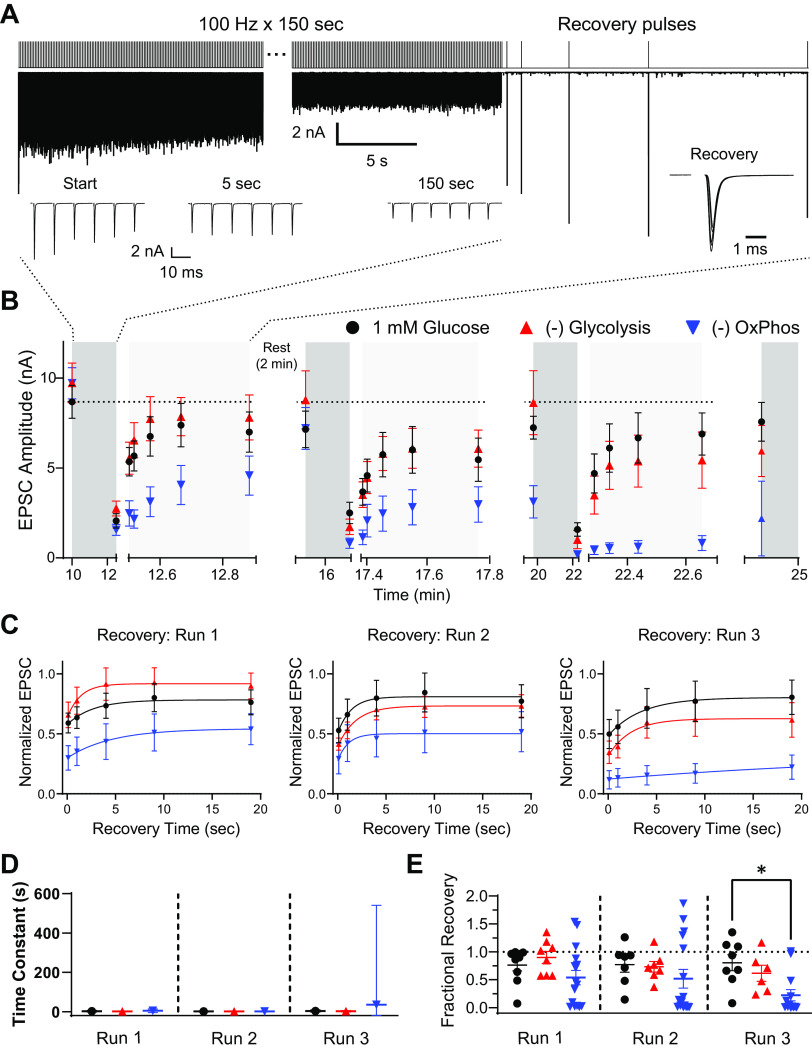
Synaptic depression and recovery due to sustained high-frequency trains after hearing onset. Sustained high-frequency stimulation (100 Hz, 150 s) was delivered, followed by single test pulses at 0.1, 1, 4, 9, and 19 s after the end of the stimulation train. *A*: stimulation protocol (*top*) and example recording (*middle*) are shown. *Insets* at *bottom* show excerpts of responses during the stimulation train illustrating synaptic depression and recovery responses from the sample recording (in 1 mM glucose). *B*: EPSC amplitudes at the beginning and end of sustained stimulation train and responses to test pulses map depression and recovery. This protocol was given three times during each recording, with 2 min rest between depression and recovery protocol (not shown). Symbols represent mean of recordings, per condition (1mM glucose *n* = 8 or 9, −glycolysis *n* = 6–8, −OxPhos *n* = 13–16). Variability in replicates reflects attenuated viability during recordings. Blocking OxPhos, but not glycolysis, resulted in deeper depression at the end of the third train [*P* = 0.0090, *F*(2, 27) = 5.638; one-way ANOVA]. *C*: time course of recovery, normalized to EPSC before sustained stimulation train, were fit with a single exponential. Recovery following the three subsequent stimulation trains are shown. Symbols are mean of recordings, per condition. *D*: comparison of recovery time constant (τ) following each sustained stimulation train. Symbols are mean of recordings per condition. Error bars are asymptotic SE of fit to mean values in Fig. 8*C*. *E*: fractional recovery of individual cells at 19 s after the end of each sustained stimulation train. Individual cells are shown. Extent of recovery was significantly impaired by blocking OxPhos after the third depression train [*P = 0.0355, *F*(8, 81) = 2.203; one-way ANOVA]. EPSC, excitatory postsynaptic current; OxPhos, oxidative phosphorylation.

In an attempt to more completely depress transmission, and more closely mimic spontaneous activity observed in vivo (51, 52), we drove activity with extended 100 Hz trains, for 150 s (2.5 min), followed by single test pulses (Fig. 8). This protocol was repeated three times, with 2 min rest between trains. Depression at the end of the first and second runs were not affected. Blocking OxPhos, but not glycolysis, resulted in deeper depression at the end of the third train (Fig. 8*B*). Recovery following these extended trains was also impaired after the third run when OxPhos was blocked (Fig. 8, *B* and *C*). Exponential fit of EPSC amplitude from test pulses, fit to the mean response across all replicates, suggest that the time constant of recovery is slowed by blocking OxPhos, especially after the third depression train where estimation confidence was also reduced (Fig. 8*D*). Extent of recovery was also significantly impaired by blocking OxPhos after the third depression train (Fig. 8*E*). Blocking glycolysis in these experiments had no effect on EPSC depression, or recovery.

**Figure 9.**
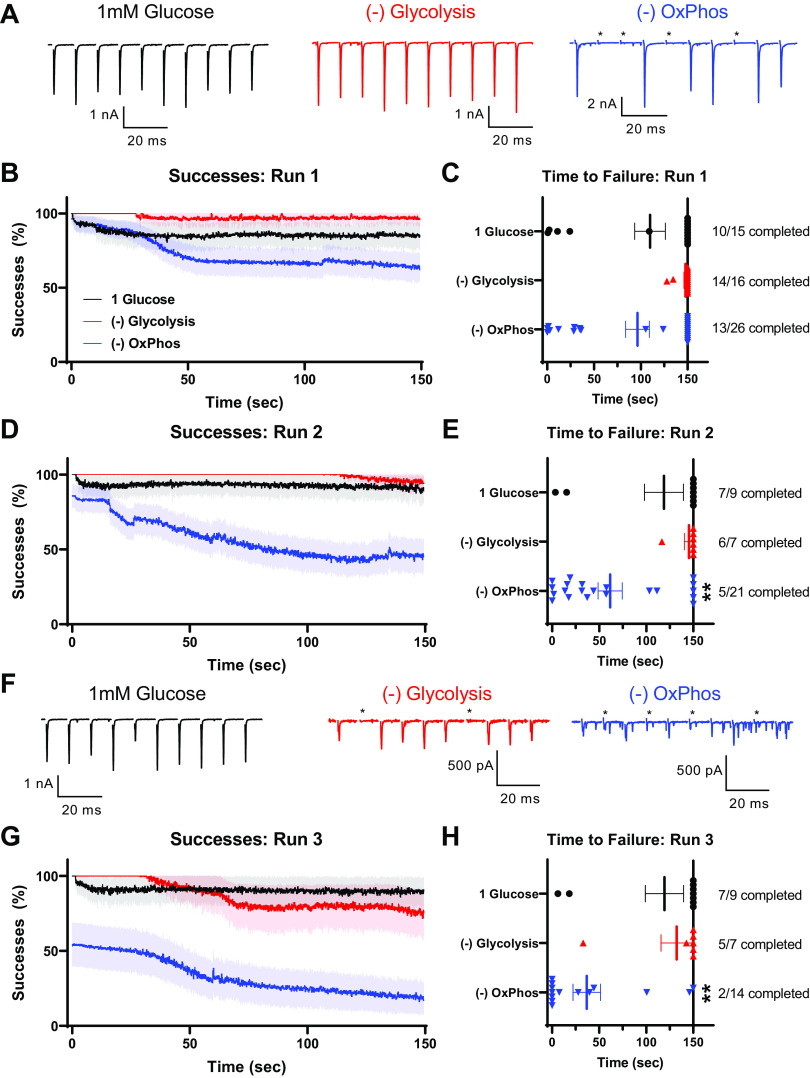
Transmission failures occur during sustained stimulation in synaptic terminals after hearing onset. Successes were recorded as events that showed a clear EPSC response relative to baseline. In most conditions, some occasional failures were observed. Data are from the same recordings as shown in Fig. 8. *A*: example recordings from the end of the first sustained stimulation train. Failures are marked with asterisks. Note large discrepancy between successful and unsuccessful events e.g., when OxPhos was blocked. *B*: successful responses plotted during sustained stimulation train, as running average of successes per 25 stimuli. *C*: time to first failure per cell for the first sustained stimulation train. “Failure” was defined as two subsequent stimuli without a corresponding postsynaptic EPSC. Symbols are results of individual recordings. Numbers to right of plot illustrate fraction of recordings that showed no subsequent failures. *D*: successful responses plotted during the second sustained stimulation train, as running average of successes per 25 stimuli. *E*: time to first failure per cell for the second sustained stimulation train. Numbers to right of plot illustrate fraction of recordings that showed no subsequent failures. [**P = 0.0572, −OxPhos vs. 1 mM glucose, *F*(8, 115) = 6.406; one-way ANOVA]. *F*: example recordings from the end of the third sustained stimulation train. Failures are marked with asterisks. Note increase in spontaneous activity when OxPhos was blocked. *G*: successful responses plotted during the third sustained stimulation train, as running average of successes per 25 stimuli. *H*: time to first failure per cell for the third sustained stimulation train. Numbers to right of plot illustrate fraction of recordings that showed no subsequent failures. *P = 0.0529, −OxPhos vs. 1 mM glucose, *F*(8, 115) = 6.406; one-way ANOVA. Transmission was slightly more successful in −glycolysis than in 1 mM glucose, and failure time was similar during the third run [*P* = 0.6413 −glycolysis vs. 1 mM glucose; one-way ANOVA]. EPSC, excitatory postsynaptic current; OxPhos, oxidative phosphorylation.

We examined traces for transmission failures, similar to the approach taken in Fig. 4 for immature synapses. Transmission was largely successful throughout all trains in 1 mM glucose and when glycolysis was blocked, but was progressively impaired when OxPhos was blocked (Fig. 9). Illustrative traces indicate that failures were likely due to impaired AP propagation, as transmission before and after failures was robust, and composed of many SV quanta, arguing against loss of vesicular supply (Fig. 9, *A* and *C*). Impairment due to loss of OxPhos was first evident after ∼30 s of stimulation (Fig. 9*A*), though two subsequent failures did not appear until ∼100 s, similar to 1 mM glucose. Notably, when OxPhos was blocked, transmission did not recover appreciably during rest intervals (Fig. 9, *B* and *C*). Blocking OxPhos resulted in strong decreases in time to failure, determined as the time when two subsequent failures occurred. At the end of the second train, sequential transmission failures occurred at about a minute during stimulation (Fig. 9*E*), and in less than 45 s during the third train, on average (Fig. 9*H*). However, some cells were able to maintain transmission until the stimulation protocol was finished (2/14 cells, Fig. 9*H*). Blocking glycolysis did not affect transmission until the third train, where some failures were observed. In fact, transmission was slightly more successful when glycolysis was blocked than in 1 mM glucose, and failure time was similar during the third run. It is unclear if this difference is due to sampling error or a result specific to forcing the synapse to depend on OxPhos for energy production.

### Modeling ATP Consumption during Sustained Activity

**Figure 10.**
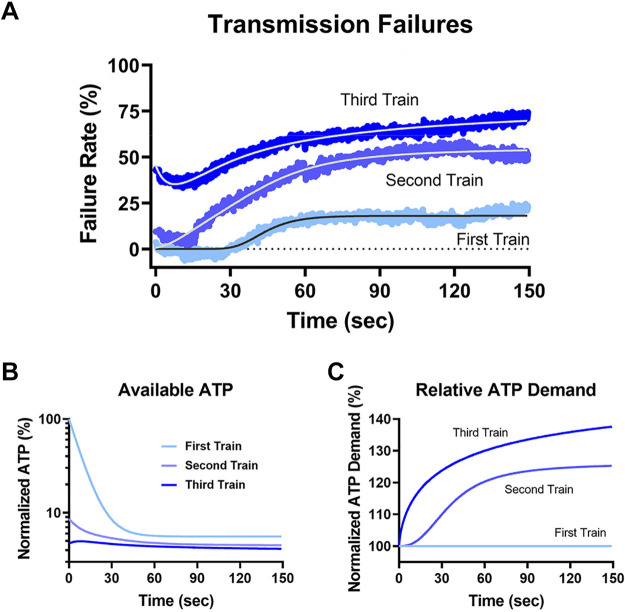
Modeling ATP demand during sustained stimulation trains. A model was generated to fit experimental data describing transmission failures following OxPhos blockade, and used to estimate ATP demand at rest and during activity. *A*: transmission failure rate was determined for the three sustained stimulation trains by subtracting success rate when OxPhos was blocked, vs. successes in 1 mM glucose. Experimental data from the three runs (symbols) were fit with a differential equation model of ATP consumption (lines), per stimulation train. *B*: estimated available ATP level during sustained stimulation trains, relative to rest before trains stimulation. ATP decayed monoexponentially with activity during the first train, and declined more rapidly during additional stimulation trains. *C*: estimated ATP demand required to sustain transmission, normalized to demand during the first train. Note that demand increases supralinearly with subsequent stimulation trains. OxPhos, oxidative phosphorylation.

Clearly, blocking OxPhos limits transmission and AP impairment seems to be an obvious measurable point of failure. In an attempt to correlate loss of transmission with loss of ATP availability and determine how ATP demand may change over time during sustained activity, we modeled transmission failures due to loss of OxPhos (Fig. 10). We converted transmission successes from Fig. 8 to failures due to OxPhos-dependent processes by subtracting summary data in the −OxPhos condition from 1 mM glucose control data during each stimulation train (Fig. 10*A*). Because we saw no loss of transmission when glycolysis was blocked in these experiments, we limited our analysis to the −OxPhos condition. We chose to use a differential equation that accounted for basal and activity-related ATP consumption, which adequately matched the experimental data from the sustained stimulation trains. No constraints or assumptions were made regarding underlying mechanisms, or absolute ATP concentration. This approach yielded information regarding relative ATP level, relative ATP demand, and relationship between available ATP and failures due to OxPhos during the three stimulation trains (see materials and methods).

It is obvious that failure rate progressively increased during the three trains and did not recover much during rest periods. During the first stimulation train, our model implies basal consumption at the calyx terminal is ≈ 0.0065 s^–1^ and increases to ≈ 0.11 s^–1^ during the first stimulation train. These estimates reveal a ≈17-fold increase in energy consumption between stimulated and basal conditions, very similar to a recent report examining Drosophila glutamatergic synapses (53). Our analysis also reveals high cooperativity between [ATP] and transmission failures (Hill coefficient *h *≈* *6), such that ATP levels must fall to 4.6% of their basal levels to reach a 50% failure rate. Failures did not occur during the first ∼30 s of stimulation of the first train, though ATP levels dropped precipitously. This result suggests a significant ATP reserve or buffer exists at this presynaptic terminal to facilitate AP firing over this initial time period.

While a constant activity-dependent consumption rate during the first train is sufficient to provide a good fit to the experimentally measured increase in failures, an additional time-varying maximal ATP consumption rate had to be incorporated to fit the results of the second and third trains, suggesting ATP demand increased beyond that in the first train due to subsequent stimulation. This additional term was used to determine available ATP levels at the start of the train, as well as fit the model to failures data for the second and third trains. For example, at the beginning of the second train, ATP availability was only 10% of basal levels, even after 2 min rest period between trains (Fig. 10*B*). ATP demand at the end of the second train increased an additional 26% over demand during the first train (Fig. 10*C*). At the start of the third train, ATP levels were estimated to be ∼4.7% of the initial level, matching an initial failure rate of ∼50%. ATP consumption in the third train increased an additional 14% over the second train, to 40% above activity-dependent consumption during the first train (Fig. 10*C*). The results of this relatively unconstrained model point to an accumulation of activity-dependent processes that must be fueled specifically during sustained high-frequency activity, and depend exclusively on acceleration of OxPhos under normal conditions.

### Loss of Transmission Is Accompanied by Multiquantal Spontaneous Release

**Figure 11.**
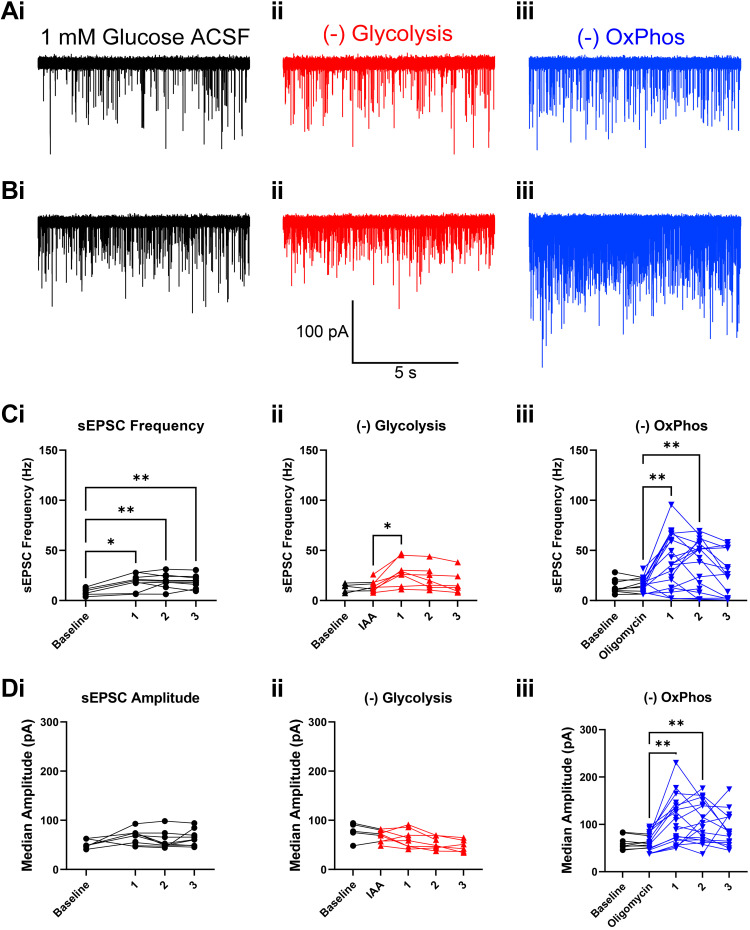
Spontaneous activity is increased after sustained stimulation in mature calyx of Held terminals. Spontaneous events recorded during the 20 s recovery phase after sustained stimulation in mature synapses (Fig. 7*A*) were recovered and separated from test pulse responses. *A*: example traces showing spontaneous EPSCs (sEPSCs) before stimulation. Frequency and size suggest they are largely composed of quantal events. *B*: example traces recovered after the first sustained stimulation train. Scale bar is shared between all traces. *C*: sEPSC event frequency prior to treatment (baseline), 10 min after treatment but before stimulation, and after sustained stimulation trains (runs 1–3). Symbols represent individual recordings, connected by lines. 1mM glucose *n* = 6–10 cells, −glycolysis *n* = 5–8 cells, −OxPhos *n* = 9–21 cells. Variability in number of replicates reflects different experimental protocols and attenuation of viability during recordings. sEPSC frequency was increased significantly in 1 mM glucose after each train, compared with activity at rest [*P* = 0.0012, *F*(1.907, 13.35) = 12.00; one-way ANOVA followed by Dunnett multiple-comparisons test]. Following sustained stimulation, smaller and transient increases were seen in −glycolysis, only significant after the first stimulation train [*P* = 0.0083, *F*(1.764, 9.704) = 8.569; mixed effects ANOVA followed by Dunnett multiple-comparisons test]. A large increase in sEPSC frequency was observed in −OxPhos after the initial stimulation trains, which diminished after the second train [*P* = 0.0170, *F*(0.8314, 10.39) = 8.687; mixed effects ANOVA followed by Dunnett multiple-comparisons test]. *D*: median sEPSC amplitudes recorded during the recovery phase after sustained stimulation. Shown are events before treatment (baseline), 10 min after treatment but before stimulation, and after sustained stimulation trains (runs 1–3). Symbols show median sEPSC amplitude per cell, connected by lines. Median sEPSC amplitude was not significantly changed by stimulation in 1 mM glucose [*P* = 0.1039, *F*(2.049, 14.34) = 2.650] or when glycolysis was blocked [*P* = 0.0822, *F*(1.223, 6.728) = 4.032], but was significantly increased when OxPhos was blocked [*P* = 0.0306, *F*(0.5712, 7.140) = 8.587; mixed-effects ANOVA]. EPSC, excitatory postsynaptic current; OxPhos, oxidative phosphorylation.

Careful examination of the traces at the end of the third train showed increased asynchronous release when OxPhos was blocked (Fig. 9*F*). Because we were able to record postsynaptic responses without concerns of receptor desensitization in older animals at near-physiological temperature (29), we examined excitatory postsynaptic currents due to spontaneous activity (sEPSCs). In a subset of cells where long 100 Hz trains were delivered, sEPSCs were captured before the first stimulation train, and during recovery periods (see example in Fig. 8*A*) in recordings from *P16–18* mice. An increase in sEPSC events was seen after the trains, as may be expected due to significant Ca^2+^ influx (Fig. 11). sEPSC frequency was increased significantly in 1 mM glucose after each train, compared to spontaneous activity at rest (Fig. 11*Ci*). Blocking glycolysis or OxPhos at rest, before stimulation trains, had no effect on sEPSC frequency (Fig. 11, *Cii* and *iii*) consistent with a marginal increase in cytosolic Ca^2+^ (Fig. 7*B*). Following sustained stimulation, smaller and transient increases were seen when glycolysis was blocked, only significant after the first stimulation train. Blocking OxPhos resulted in a large increase in sEPSC frequency after the initial stimulation train, which diminished after the second train. sEPSC event frequency was no longer elevated after the third train when OxPhos was blocked. Notably, the dispersion of spontaneous event frequency was substantially increased by blocking OxPhos (CV = 0.70 after 1st train), versus glycolysis (CV = 0.25) or in 1 mM glucose (CV = 0.36).

As dispersion and size of events seemed to change after stimulation trains, especially when OxPhos was blocked, we examined sEPSC amplitudes (Fig. 11*D*). Compared with sEPSCs at rest, median sEPSC amplitude was not significantly changed by stimulation in 1 mM glucose or when glycolysis was blocked, but was significantly increased when OxPhos was blocked. This increase in median sEPSC size for −OxPhos was present after the first and second trains, but absent in the third train (Fig. 11*Diii*). On the whole, these results indicate that loss of transmission when OxPhos is blocked may be due to a combination of AP failure, potentially accompanied by a breakdown in regulated SV release and recycling, leading to increases in unregulated SV release.

## DISCUSSION

Brain function is dependent on constant availability of glucose and oxygen delivery from the bloodstream. Acute hypoglycemic or ischemic conditions quickly result in loss of chemical synaptic transmission due to loss of presynaptic function (54, 55). Although most neuronal ATP is produced by mitochondria (1), the role of glycolysis is less well appreciated. We addressed whether and when glycolysis or OxPhos may be preferred, or required, to maintain excitatory transmission. Local availability of energetic substrates, temporal demands during synaptic activity, and synapse type or developmental stage could all have an effect on fuel selection. We tested whether neuronal metabolism at synapses in acute brain slices mimicking physiological conditions (temperature, Ca^2+^, and glucose concentrations) may behave differently from cultured neurons or brain slices maintained in hyperglycemic conditions.

This study examined the relative contribution of glycolytic- versus mitochondrially derived ATP to support presynaptic function during high-frequency neurotransmission, in a synapse specialized for sustained high-frequency firing. Using two well-characterized pharmacological inhibitors, we selectively inhibited either glycolysis (with iodoacetic acid), or mitochondrial respiration (with the ATP synthase inhibitor oligomycin), leaving the other pathway for ATP production intact. To our surprise, there was little activity-dependent effect of inhibiting either pathway during brief stimulation trains beyond a previously described effect on initial SV release (10), and failures were only apparent in response to sustained high-frequency stimulation.

### Short Trains Are Not Impacted by Selective Block of Glycolysis or OxPhos

We saw very little impairment in transmission due to short repetitive bursts of activity during 20 min of stimulation in recordings, in both immature and mature synapses, when either glycolysis or OxPhos were selectively blocked, suggesting significant redundancy between the two modes of ATP production and/or a significant energy reserve in the presynaptic terminal during these transient increases in activity. At the calyx of Held, as with most synapses, initial priming and RRP refilling is dependent on ATP (44, 56). Loss of presynaptic ATP slows the initial rate of SV mobilization from a recycling pool or a prematurely primed pool to a readily releasable state. Because we saw only minor defects in transmission in these protocols, we assume that ATP levels were maintained. Of course, blockade of both glycolysis and OxPhos resulted in loss of transmission after ∼15 min in the absence of activity, though labile cytosolic Ca^2+^ increased beginning at ∼5 min after exposure (Fig. 7). As expected, when mature synapses were stimulated transmission failed rapidly and did not recover, confirming that the terminal requires new ATP production and that iodoacetic acid and oligomycin are effective blockers of energy production.

These results are consistent with recent studies that suggest extracellular monocarboxylates (via OxPhos) or glucose (via glycolysis) are sufficient to support synaptic function (15, 57–59). In the absence of mitochondrial respiration, coordinated pathways within neurons are activated that compensate for apparent ATP decrements. The glycolytic “metabolons” have been proposed to maintain proper presynaptic function during hypoxia in *Caenorhabditis elegans*, whereas mitochondrial inhibition leads to preferential insertion of glucose transporters to the plasma membrane in cultured hippocampal neurons (8, 60). The mature calyx of Held uses similar metabolic plasticity, as transmission can be maintained in the presence of either glucose or lactate during stimulation up to 30 s (17).

### Developmental Shift in Energetic Substrate Utilization

Prolonged trains of stimulation, similar to and longer than those used in Lucas et al. (17), did result in transmission failures but required several bouts of activity. Here, we did see a developmental shift in glycolysis versus OxPhos utilization. This shift is in line with a host of developmental changes that occur during postnatal development. During the first month after birth, the calyx synapse undergoes massive molecular and structural changes to support high frequency, high fidelity transmission (reviewed in Ref. 50). Many of these presynaptic changes improve metabolic efficiency. Low SV release probability and extremely short AP waveform, for example, act to reduce metabolic load during repetitive activity. Tighter coupling of SVs to voltage-gated calcium channels and reduction of Ca^2+^ influx to small nano-domains at the developmentally mature calyx (61, 62) will reduce the energetic load on the plasma membrane Ca^2+^ ATPase (PMCA) for ion extrusion. An increase in synaptic Na/K ATPase and mitochondria also occur during this developmental period (22, 63). Our results suggest that a developmentally regulated change in the metabolic profile exists at the calyx of Held, as glycolysis was required to maintain transmission before hearing onset (Figs. 3 and 4), whereas synapses from animals after hearing onset were only perturbed by blocking OxPhos, with no effect of blocking glycolysis (Figs. 8 and 9).

A potential mechanism supporting the specific utilization of monocarboxylates in mature terminals is indicated by the astrocyte-neuron lactate shuttle hypothesis (64). In brief, this hypothesis suggests that astrocytes provide neurons with monocarboxylates (e.g., lactate), which can be imported and utilized as fuel for OxPhos. Although controversial, recent work shows that the mature calyx terminal can utilize extracellular lactate when glucose is limited, via uptake through monocarboxylate transporters (17). Efficient operation of this shuttle requires close apposition of astrocyte processes near the calyx terminal. Glia are present early in development of this synapse (65, 66) and become highly interdigitated with the mature terminal (67). Surprisingly, our data suggest that in mature terminals energy production is exclusively reliant on exogenously supplied monocarboxylate substrates to fuel OxPhos and support function. Lack of this mechanism in younger animals may be due to an absence of monocarboxylate transporters in neuronal membrane at the presynaptic terminal. Future work should focus on determining the expression pattern of these transporters during development.

### Sustained Transmission Impairs Synaptic Transmission Due to Action Potential Loss and Impaired Synaptic Vesicle Recycling

At the mature terminal, sustained high-frequency trains mimicking endogenous spontaneous activity at this synapse showed defects in transmission after ∼30 s when OxPhos was blocked, precipitated by loss of AP propagation (Fig. 9). Following additional long stimulation trains, defects in synaptic recovery (Fig. 8) and disrupted quantal size and release (Fig. 11) were observed. We infer AP failures occurred during extended stimulation trains due to stochastic loss of transmission (examples in Figs. 4 and 9). We did not observe any effect of inhibited OxPhos on the AP shape when the synapses were at rest in an earlier study (10). AP failures were not observed in a recent paper using the same synaptic preparation but shorter duration trains (17). Minor technical discrepancies including larger stimulation voltages could account for this difference, though transmission failures were not rescued with increased voltages in our experimental setup. Consistent with both sets of data, we suggest that an ATP reserve exists to support high-frequency transmission up to 30 s, with increased ATP demand when stimulation persists beyond that time. That the train durations used in Lucas et al. (17) did not elicit failures suggest that a threshold for increased ATP demand exists, fueled specifically by OxPhos. Reports using primary neuronal cell cultures have not reported AP failures, though field stimulation in those preparations do evoke APs (68). Alternatively, oligomycin inhibition could impair SV fusion independent of ATP maintenance by decreasing a-ketoglutarate (69); however, the all-or-none loss of transmission and stochastic nature of failures, but no effect on synaptic depression, argues against this mechanism.

Intracellular recordings from the terminal could be used to measure AP waveform during activity, but these recordings are very challenging. Subsequent dialysis with intracellular recording media requires perforated patch recording for extended time periods (20+ min) and rigorous stimulation conditions. On-cell recordings of the pre-AP waveform are also not possible at physiological temperature in this preparation, as the AP waveform is embedded in the stimulus artifact. Though indirect, our results show that presynaptic AP failures are possible, and likely dependent on mitochondrial respiration in mature synapses, but occur only after prolonged high-frequency activity at the calyx of Held.

In older animals, loss of OxPhos also resulted in increased spontaneous activity, and altered event amplitudes, including a potential increase in multiquantal release (Fig. 11). We also observed decreases in effective quantal size after activity when either glycolysis or OxPhos were blocked, indicating SV refilling during rapid recycling may be impaired. Altered quantal size due to oligomycin treatment could be a downstream effect of oligomycin inhibition decreasing metabolic precursors for glutamate production, as well as limiting ATP necessary for vesicle filling with transmitter. Increased labile cytosolic Ca^2+^ in the presynaptic nerve terminal following prolonged activity is a likely cause for the increase in sEPSC frequency we observed after sustained stimulation, and could partially explain multiquantal release. At the calyx of Held, relatively small (<2 µM) influxes of Ca^2+^ during synaptic activity are cleared from the presynaptic nerve terminal primarily by ATP-independent exchangers, and engage the PMCA following higher [Ca^2+^]_cyto_ load (70). PMCA2 is strongly expressed both pre- and postsynaptically at the calyx of Held (71). Thus, one attractive mechanism for increased cytosolic Ca^2+^ is impaired PMCA function when OxPhos is blocked. However, this interpretation stands in contrast to previous results in dendrites and somatic compartments from cerebellar neurons, using an imaging-based approach (36). Cytosolic Ca^2+^ accumulation and extrusion via PMCA in these cells was shown to be dependent solely on glycolysis, with no impact of inhibiting mitochondrial ATP synthase with oligomycin. Additional studies will be needed to explore this potential mechanism.

### Sustained Activity Increases ATP Demand Supralinearly

During these prolonged stimulation trains, modeling indicates ATP demand increases supralinearly with respect to activity. Although our model does not imply any specific mechanism, at least three distinct processes must be accommodated. First, PMCA-dependent clearance of Ca^2+^ from the presynaptic terminal will increase as endogenous buffers and Ca^2+^ exchangers are overwhelmed (70). Second, translocation of SVs from a reserve pool to recycling pool is ATP- and activity-dependent (72–74). Third, recycling of SVs is also highly energetic, but generally slower relative to the first two processes. In addition, SV retrieval is slowed by increased cytosolic Ca^2+^, which will accumulate during sustained stimulation (75, 76). Accumulation of material at the presynaptic active zone will require unraveling of cis-SNARE complexes, membrane and protein sorting, eventual biogenesis of new SVs, and are thus likely consumers of ATP (77). Notably, none of these processes affect AP propagation directly, but may reduce presynaptic ATP levels below those needed to power the Na/K membrane pumps and maintain ionic balance at the presynaptic terminal.

To offset increased demand, presynaptic mitochondrial Ca^2+^ uptake stimulates respiration and ATP production, but requires substantial accumulation of Ca^2+^ in the mitochondrial matrix (58, 78). Mitochondrial Ca^2+^ increases rapidly at the mature calyx within a few stimuli (79, 80). We assume that during sustained stimulation used here the threshold for increased respiration is met, facilitating OxPhos production of ATP. An alternate explanation to explain the delayed effect of OxPhos blockade on transmission, an effective ATP buffering mechanism may be present in the calyx terminal to readily distribute high-energy phosphates to support SV recycling pathways. For example, local ATP regeneration can be managed by the phosphagen system (81). Additional experiments will address the role of ATP buffering and local regeneration in this presynaptic terminal.

### Comparison to Other Excitatory Synapses

Our result stands in partial contrast to other recent reports using primary neuronal cultures, which suggest loss of mitochondrial ATP immediately compromises transmission, by inhibiting SV recycling of the readily releasable pool and impairing endocytosis (2, 15, 16). Other recent work shows that network activity is dependent on glycolysis in cultured neuronal preparations and cannot be substituted for by monocarboxylate substrates (82); however, cell viability is maintained in the absence of activity for prolonged periods of time due to low circulating levels of lactate (18). We believe these differences may be due to the synapse type examined (hippocampal versus auditory brainstem) and other experimental conditions (neuronal culture versus acute brain slices). Our results are consistent with reports showing a division of energetic support in neurons, where glycolysis supports basal transmission and high-frequency transmission is more reliant on mitochondrial respiration (14, 16). Specifically, we propose that the mature calyx synapse is resistant to rundown due to its massive SV recycling pool size. As a result, prolonged sustained activity is necessary to observe a defect in transmission.

In conclusion, we find either OxPhos or glycolysis can support transmission during short bouts of activity at the calyx of Held. During sustained high-frequency activity, glycolysis is predominantly required for synaptic function in immature terminals, whereas presynaptic function is nearly completely dependent on OxPhos in mature animals. These metabolic adaptations add to our understanding of the host of refinements supporting high frequency, high fidelity transmission at this synapse.

## GRANTS

This study was supported by National Institutes of Health under Grants GM103554 and NS117686 (to R.B.R.), DC019268 (A.S.), and National Science Foundation Grant 1943514 (to R.R.). B.J.L. was supported by the Michael (Mick) J.M. Hitchcock, Ph.D. Fund.

## DISCLOSURES

No conflicts of interest, financial or otherwise, are declared by the authors.

## AUTHOR CONTRIBUTIONS

B.J.L., A.S., and R.B.R. conceived and designed research; M.S., A.S., and R.B.R. performed experiments; B.J.L., A.S., and R.B.R. analyzed data; R.B.R. interpreted results of experiments; B.J.L., A.S., and R.B.R. prepared figures; B.J.L. and R.B.R. drafted manuscript; M.S., A.S., and R.B.R. edited and revised manuscript; B.J.L., M.S., A.S., and R.B.R. approved final version of manuscript.
